# Advanced Research in Forensic Genetics

**DOI:** 10.3390/genes17050582

**Published:** 2026-05-19

**Authors:** Mauro Pesaresi, Alessia Bernini Di Michele

**Affiliations:** Section of Legal Medicine, Department of Biomedical Sciences and Public Health, Polytechnic University of Marche, Via Tronto, 60126 Ancona, Italy; a.bernini@pm.univpm.it

This Special Issue on advanced research in forensic genetics aims to showcase recent methodological developments, emerging challenges, and future perspectives in a field that is rapidly evolving under the pressure of technological innovation and increasing interpretative complexity. Modern forensic genetics encompasses population genetics, kinship analysis, DNA transfer dynamics, complex and degraded sample analysis and predictive approaches such as forensic DNA phenotyping. The contributions collected in this Special Issue reflect this multidimensional evolution, emphasizing both the opportunities and the critical need for robust interpretative frameworks. This Special Issue brought together six original research articles and two reviews that collectively highlight both the advances and persistent challenges in contemporary forensic genetics.

At the population level, K. Alkaraki et al. [1] provided a clear example of how genetic markers can inform both forensic databases and anthropological interpretation. By analyzing Y-chromosome variation in Jordanian Bedouin and Fellahin groups, the authors demonstrated how social structure, mobility, and cultural practices shape paternal genetic diversity.

A similar concern with interpretation emerges in the work of Kruijver [2], which focused on identity-by-descent (IBD) segments to distinguish biological relationships. This study investigated the evidential value obtained for discriminating between common pedigree relationships if IBD is observed continuously across the autosomal genome without error. In the continuous case, the evidential value is limited only by the pedigree relationship and recombination rates. This highlighted a recurring issue in forensic genetics: as analytical sensitivity increases, so does the complexity of interpreting results within a probabilistic framework.

Another complex field in forensic genetics concerns DNA transfer. The experimental study by Lee et al. [3] demonstrated that secondary DNA transfer between items stored in the same evidence package is far from negligible. Even without direct contact, DNA can transfer in a significant proportion of cases, sometimes influencing forensic interpretation.

These findings align with the broader discussion presented in the review by Bini et al. [4], which examined the role of domestic animals as vectors of human DNA. Pets can act as reservoirs and intermediaries of genetic material, enabling both primary and secondary transfer across multiple steps. Together, these studies emphasize that DNA evidence cannot be interpreted in isolation from mechanisms of transfer and the context in which biological traces are deposited.

The challenge of interpreting DNA evidence is compounded when dealing with complex or degraded samples. The contributions focusing on formalin-fixed, paraffin-embedded (FFPE) tissues illustrated the technical limitations of current methodologies (Lisman et al. [5]). Although improved extraction kits, such as the Maxwell^®^ RSC Xcelerate system, allowed the recovery of relatively high quantities of DNA, the quality of the genetic material often remained insufficient for complete STR profiling. These findings underlined the need for alternative approaches, such as miniSTRs, SNP panels, and next-generation sequencing, particularly in cases where FFPE samples represent the only available source of DNA.

The use of FFPE tissue is also common in post-mortem analyses, particularly when these blocks represent the only available biological material. This is illustrated by the study conducted by Bernini et al. [6], which investigated genetic variation by analyzing both blood and FFPE tissue samples using NGS techniques. The aim was to identify variants in cardiac genes associated with sudden unexpected death in epilepsy (SUDEP).

Another highly studied topic in advanced research in forensic genetics is forensic DNA phenotyping (FDP). The review by Sessa et al. [7] outlines the current state of the art, with particular attention paid to low-template DNA and the integration of machine learning models to enhance predictive accuracy. However, the authors also stress the importance of addressing ethical considerations and population biases, reminding us that technological innovation must be accompanied by responsible implementation.

González-Ortiz et al. [8] evaluated the performance of FDP using the ForenSeq™ Imagen kit on a Mexican mestizo population. Phenotype prediction was performed using the HIrisPlex-S model, while ancestry inference relied on principal component analysis. The results showed that ancestry, hair and skin color, and sex prediction were generally robust, even under suboptimal DNA conditions. However, eye color prediction was significantly affected by the dropout of key SNPs, leading to reduced accuracy. Overall, the study highlights that FDP performance strongly depends on the presence of specific high-impact genetic markers rather than overall genotype completeness.

Taken together, the contributions in this Special Issue illustrate a field that is rapidly evolving toward greater sensitivity and sophistication. However, they also make clear that progress in forensic genetics is not solely a matter of technological advancement; equally crucial is the development of robust interpretative frameworks that account for population diversity, DNA transfer dynamics, sample quality, and probabilistic reasoning. Bridging these aspects will be essential in ensuring that DNA evidence continues to serve as a reliable and scientifically grounded tool within the justice system.

## References

[1] Alkaraki A.K., Alsliman M.B., Twait M.M., Alfonso-Sánchez M.A., Peña J.A. (2026). Genetic Diversity of 27 Y-STRs in Two Jordanian Subpopulations: Bedouins and Fellahin. Genes.

[2] Kruijver M. (2025). An Upper Bound on the Power of DNA to Distinguish Pedigree Relationships. Genes.

[3] Lee Y.S., Syn C.K.-C. (2025). DNA Transfer between Items within an Evidence Package. Genes.

[4] Bini C., Trasatti A., Giorgetti A., Amurri S., Fazio G., Pelotti S. (2026). A Mini Narrative Review on Human DNA Transfer Involving Dogs and Cats and Their Role in Forensic Investigation. Genes.

[5] Lisman D., Ossowski A., Tołoczko-Grabarek A., Kozłowski M., Cymbaluk-Płoska A. (2025). Forensic DNA Recovery from FFPE Tissue Using the Maxwell^®^ RSC Xcelerate Kit: Optimization, Challenges, and Limitations. Genes.

[6] Bernini Di Michele A., Onofri V., Melchionda F., Fiordelmondo L., Ciarimboli E., Palpacelli M., Sablone S., Turchi C., Pesaresi M. (2025). Cardiac Genetic Variants in Sudden, Unexpected Death in Epilepsy: From Challenging DNA Extraction Methods to Updated NGS Panels for Improved Genetic Analysis. Genes.

[7] Sessa F., Dervišević E., Esposito M., Francaviglia M., Chisari M., Pomara C., Salerno M. (2026). Predicting Physical Appearance from Low Template: State of the Art and Future Perspectives. Genes.

[8] González-Ortiz N., Guardado-Estrada M., Zepeta-Flores N., Moreno-Ortiz J.M., Ramírez-de-Arellano A., Rangel-Villalobos H., Muñoz-Valle J.F., Aguilar-Velázquez J.A. (2026). Performance of the ForenSeqTM Imagen Kit for Forensic DNA Phenotyping under Partial Genotyping Conditions. Genes.

